# Pet Ownership and Quality of Life: A Systematic Review of the Literature

**DOI:** 10.3390/vetsci8120332

**Published:** 2021-12-16

**Authors:** Kristel J. Scoresby, Elizabeth B. Strand, Zenithson Ng, Kathleen C. Brown, Charles Robert Stilz, Kristen Strobel, Cristina S. Barroso, Marcy Souza

**Affiliations:** 1 College of Social Work, University of Tennessee, Knoxville, TN 37996, USA; kscoresb@vols.utk.edu; 2 Veterinary Social Work, Colleges of Veterinary Medicine and Social Work, University of Tennessee, Knoxville, TN 37996, USA; estrand@utk.edu; 3 College of Veterinary Medicine, University of Tennessee, Knoxville, TN 37996, USA; zng@utk.edu (Z.N.); cstilz@vols.utk.edu (C.R.S.); kstrobel@vols.utk.edu (K.S.); 4 Department of Public Health, University of Tennessee, Knoxville, TN 37996, USA; kcbrown@utk.edu (K.C.B.); cbarroso@utk.edu (C.S.B.)

**Keywords:** pet ownership mental health, human-animal bond, human-animal interactions

## Abstract

Pet ownership is the most common form of human–animal interaction, and anecdotally, pet ownership can lead to improved physical and mental health for owners. However, scant research is available validating these claims. This study aimed to review the recent peer reviewed literature to better describe the body of knowledge surrounding the relationship between pet ownership and mental health. A literature search was conducted in May 2020 using two databases to identify articles that met inclusion/exclusion criteria. After title review, abstract review, and then full article review, 54 articles were included in the final analysis. Of the 54 studies, 18 were conducted in the general population, 15 were conducted in an older adult population, eight were conducted in children and adolescents, nine focused on people with chronic disease, and four examined a specific unique population. Forty-one of the studies were cross-sectional, 11 were prospective longitudinal cohorts, and two were other study designs. For each of the articles, the impact of pet ownership on the mental health of owners was divided into four categories: positive impact (*n* = 17), mixed impact (*n* = 19), no impact (*n* = 13), and negative impact (*n* = 5). Among the reviewed articles, there was much variation in population studied and study design, and these differences make direct comparison challenging. However, when focusing on the impact of pet ownership on mental health, the results were variable and not wholly supportive of the benefit of pets on mental health. Future research should use more consistent methods across broader populations and the development of a pet-ownership survey module for use in broad, population surveys would afford a better description of the true relationship of pet ownership and mental health.

## 1. Introduction

Throughout history, animals have played a significant role in society including in agriculture and pet ownership. A recent survey conducted in the United States estimated that approximately 67% of homes had at least one pet, equaling about 63 million homes with at least one dog and 42 million homes with at least one cat [1]. Pets can constitute a connection to nature, function in recreational and work activities, and provide companionship in our homes [2,3,4]. The importance of animals in our lives is founded on the human–animal bond concept, which is the “mutually beneficial and dynamic relationship that exists between people and other animals that is influenced by behaviors that are essential to the health and well-being of both” [5]. This concept has championed animals as companions and family members, leading to their essential part of everyday life for many. The human–animal bond has additionally driven the common belief that pets are good for human health, both physical and mental [6,7,8].

While there are some qualitative [9,10] studies that claim that pet ownership benefits people, particularly in regard to improved mental health, there are few studies with substantial evidence from large, diverse population samples to support this theory. The studies that have been published are often not substantiated with regard to study populations or methods, making broad conclusions difficult. Furthermore, some studies that have investigated the correlation between pet ownership and mental health have revealed no effect, or even worse, negative effects of pet ownership [11,12,13,14,15]. The inconsistencies in the literature and limitations of these studies warrant a thorough exploration of the effect of pet ownership on mental health outcomes among large, diverse population samples.

Two previous systematic reviews of the literature did examine the relationship between pet ownership and mental health/well-being [16,17]. Islam and Towel [16] did not find a clear relationship between pet ownership and well-being in the 11 studies included in their review. Similarly, Brooks et al. [17] examined the role of pets in owners with diagnosed mental health problems and found mixed results across the 17 studies included in the review. The purpose of this study was to perform a systematic review of the peer-reviewed published literature containing original research that examined the relationship between pet ownership and mental health for people in any population. Previous reviews included a smaller sample of research articles, often limited to a specific population of pet owners. By describing the relationship between pet ownership and mental health across all examined populations, this study will better inform whether pets could be recommended to help with mental health and whether promotion of the human–animal bond is generally beneficial.

## 2. Materials and Methods

The systematic review process involved a literature search, screening, extraction, and an assessment of the remaining articles by four researchers and three graduate students. For the purpose of this study, pet ownership was limited to dogs and cats. Our research team sought to answer, “How does ownership of a dog or cat influence the mental health or quality of life of pet owners?”

In May of 2020, the following databases were searched for peer-reviewed articles on pet ownership and mental health: PubMed and Web of Science. Utilizing Boolean search terms, the literature search was conducted using the terms: anxiety OR depressi* OR bipolar OR (mental* AND (health OR disease* OR disorder* OR condition* OR ill*) for the problem, (dog OR dogs OR cat OR cats OR canine* OR feline*) AND ((pet OR pets)) AND (owner* OR companion* OR interact* OR bond* OR “human animal bond” OR “animal human bond” OR “animal assisted”) for the intervention and health* AND (impact* OR outcome* OR status OR effect* OR affect* OR consequen* OR result*) for the outcome.

**Figure 1:**
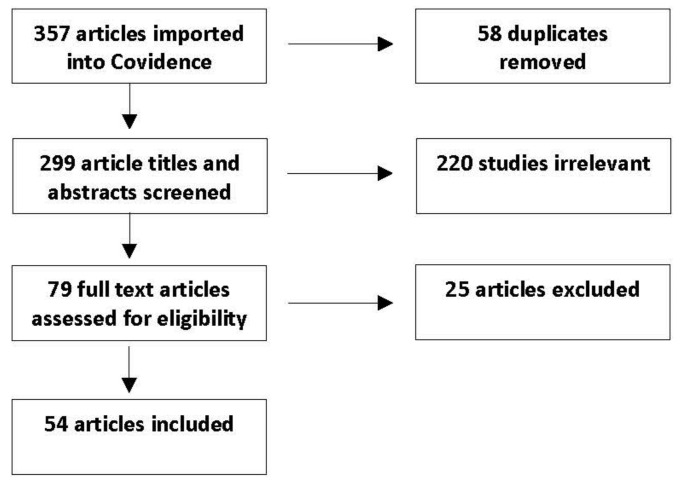
Following a literature search, articles were reviewed for adherence to inclusion and exclusion criteria. A total of 54 articles were identified to meet all criteria.

Although there was not an approved PRISMA protocol, the research team used Covidence (Melbourne, Australia), a software program that tracks the systematic review screening process. Identified articles were imported into Covidence, duplicates were removed, and the remaining articles were screened by the research team. Through random assignment, each article was independently reviewed by one faculty member and one graduate student. Each reviewer indicated in Covidence if the article should be included or excluded according to established criteria (Table 1). When there was a conflict between reviewers, a third reviewer (non-student) resolved the conflict. The full review process is shown in Figure 1. At the final review stage, two researchers independently extracted specific information (Table 2) from each article. The type of impact on mental health was determined based on the results reported in each article.

In addition to extracting the information outlined in Table 2, an index (Appendix A) was created to assess article quality. The index was based on two previous systematic reviews of mental health in veterinary science [17,18]. Each dichotomous index question assigned a 0 if the article did not meet criteria and a 1 if the article did meet criteria. The higher the score an article received (0–9 points), the higher the quality of the article.

Interventionary studies involving animals or humans, and other studies that require ethical approval, must list the authority that provided approval and the corresponding ethical approval code.

## 3. Results

The article review process and number of articles in each step are shown in Figure 1. A total of 54 articles met the inclusion and exclusion criteria (Table 1) and were systematically extracted (Table 2). These articles were then divided into four categories based on the type of overall impact pets had on the mental health of owners: (1) positive impact (n = 17); (2) mixed impact (*n* = 19); (3) no impact (*n* = 13); and (4) negative impact (*n* = 5). Factors that influenced mental health include (a) age (middle-aged female caregivers had more psychological stress than young female and male caregivers), (b) obedience and aggressiveness of the pet, (c) marital status (single women who owned a dog were less lonely and socially isolated than women without pets), and (d) attachment to the pet (high level of bonding has lower anxiety and depression scores than lower level of bonding) [19,20,21,22,23,24]. A few representative studies with mixed results include one examining the general population, which found that unmarried men who live with a pet had the most depressive symptoms and unmarried women who live with a pet had the fewest [19]. Another study examining the impact of companion animals on cancer patients found that mental health was associated with the status of cancer treatment, with those receiving intense treatment having poorer mental health [20]. In addition to overall impact, the study population, study type, population size, year of publication and article quality are reported (Appendix B).

Of the 54 articles, 19 (35%) were studies conducted in the general population, 15 (28%) were studies in older adult individuals, eight (15%) were in children and adolescents, six (11%) focused on people with some type of chronic physical illness/disease, three (6%) were studies in people with severe mental illness, and three (6%) studies examined unique populations. Of the 15 studies that had only older adult participants, none of them reported a positive impact. Seven of the articles reported mixed impact based on type of pet, gender, companionship, or another demographic. Six of the studies had no impact and two had a negative impact. Of the eight studies that involved children and adolescents, six of them indicated a clear positive impact, one indicated mixed impact, and one indicated no impact. Of the three studies that involved those with severe mental illness, two indicated clear positive impact and one indicated mixed impact.

Research studies either compared mental health outcomes in pet owners versus non-pet owners (*n* = 41) or with regard to owner attachment to the pet (*n* = 13). Similar to the overall distribution, the outcomes within these two different types of studies were distributed across all four categories (Table 3 and Table 4). In 38% (five of 13) of the studies, attachment to a cat or dog was associated with a positive impact on mental health in 38% of the studies. Four of the 13 studies (31%) indicated mixed results, meaning that human–animal attachment sometimes was associated with better mental health and sometimes it was not. One example of higher attachment leading to worse mental health was for those amid cancer treatment [20]. There was no clear trend towards attachment and better mental health.

The study types included 41 (76%) cross-sectional studies, 11 (20%) prospective cohort longitudinal studies, and two (4%) other study designs. Of the cross-sectional studies, 27 (66%) found that companion animals had no or negative impact on mental health and 14 (34%) found mixed or positive impact on mental health. Of the 11 articles that reported on a longitudinal study design, five (45%) demonstrated no or negative impact and six (55%) demonstrated mixed or positive impact. Among the 54 studies, sample size ranged from 30 to 68,362.

To measure mental health constructs, 75 different validated scales were used (Table 5). Eight scales were used to measure human attachment to pets. The most common scales used across studies were the CES-D (13 studies) to measure depression and the ULS (10 studies) to measure loneliness. Two scales were used by four studies each (DASS and any variation of GHQ). Three scales were used by three studies each (GDS, CABS, and any variation of PHQ). The remaining scales were used only once or twice across the studies assessed.

Regarding the study quality scores (Appendix A), no articles received a quality score of 9, six (11%) received a score of 8, 11 (20%) received a score of 7, 20 (37%) received a score of 6, and 17 (31%) received a score of 5 or below. Of the articles with a quality scale score of 5 or lower, 18% (3) articles had no or negative impact and 82% (*n* = 14) had mixed or positive impact on owner mental health. Articles with a quality scale score of 6 or higher, 43% (*n* = 16) showed no or negative impact and 57% (*n* = 21) showed mixed or positive impact.

## 4. Discussion

Understanding the nature of the relationship between mental health and pet ownership is important for both human and animal welfare and to better determine the impact of human–animal interactions. Over the years, the perspective that “pets are good for you” has become an assumption [25] and when negative implications are recognized it often relates to zoonotic diseases rather than human–animal interactions [26]. This belief in the positive aspects of the human–animal bond is strengthened by marketing tools used by the pet industry [27]. While there certainly is evidence that supports the benefits of the human–animal bond to people’s mental health [28,29], there is also clear and consistent evidence that the relationship is complex and sometimes negative [30,31]. The question of whether pets should be prescribed by health professionals is an especially important one. Recent qualitative research supports that attending to a pet can help a person manage mental health crises [32], however, doing so can also cause a person to rely on the pet instead of other evidenced based methods of seeking mental health support. The recommendation of obtaining a pet in the presence of mental illness ought to be coupled with other evidenced based strategies for mental health recovery such as increasing social support and engaging in third wave behaviorally based interventions such as Acceptance and Commitment Therapy or Dialectical Behavior Therapy.

The broad perspectives that pets are good for mental health may cause people to place false expectations on the role a dog or cat must play in their lives [33]. The anthropomorphism of pets (people placing human cognitive motivations on pets’ behavior and treating pets as people) can in fact have a negative impact on the animal’s welfare [34]. The untreated stress of people who turn to their pets instead of their human social supports and health professionals may in fact be causing pets to be more stressed [35]. Although initial data suggest relinquishment rates were not higher after COVID-19 lockdowns were lifted [36], some still have concerns that the recent increase in pet adoptions from shelters may result in pet relinquishment once the pandemic is more managed and people return to their daily work environments [37] (J. Schumacher personal communication, 5 May 2021). Developing clear guidelines about the benefits and liabilities of pet ownership and mental health is important to mitigate the public halo effect that suggests that simply acquiring a pet will improve your mental health.

Previous systematic reviews of the literature have found mixed results regarding the relationship between mental health and pet ownership [16,17]. Our search and review methodology was similar to Islam and Towel [16], which yielded 11 studies compared to the 54 studies compiled in this review. Although the Brooks et al. [17] review yielded 17 studies, they limited their search to studies only including people diagnosed with mental health conditions. While the current study did examine a larger body of research that covered broader populations and more recent publications than previous reviews, the findings were similar in that results varied across outcomes including positive, negative, mixed, and negligible. Unlike previous studies, this review also differentiated studies that compared pet owners to non-pet owners and studies that examined the level of attachment with a pet as a predictor of the mental health of the owner. Islam and Towel [16] argued that the definition of pet ownership needs to be defined across all studies, including aspects of length of ownership, time spent with the animal, and perceived quality of the interaction. Within these two categories of study types, the outcomes still varied and showed no consistent evidence that pet ownership is a positive contributor to mental health. The lack of consensus from these studies was not surprising. While popular literature and media consistently highlight the positive, it rarely highlights the negative aspects of pet ownership. In fact, studies with negative or non-significant findings are often subject to the “file drawer” effect, in which authors ultimately decide not to publish their studies [15]. In this review, we did find and include studies that reported negative or mixed findings.

The authors made the decision a priori to divide the results into categories based on the type of impact each study had on mental health. Among the 17 studies that were determined to have positive results, most of the studies were with children and adolescents (*n* = 6) and the general adult population (*n* = 6). There were some challenges to identifying these studies as clearly positive. Because a variety of different variables and a variety of different methodologies were used based on the specific purpose of each study, they could not be directly or easily compared to one another. Many of the positive impact studies investigated additional variables that could be better predictors of positive mental health than dog/cat ownership. For example, several studies indicated that children or adolescents with a dog had less depression and/or less anxiety than peers without a dog. However, family dynamics such as single parent or two parent households, time parents spend at work, presence of siblings, and family dysfunction [2,8] may be more significant contributors to child mental health than dog ownership.

The 19 mixed impact studies were easier to categorize because of conflicting outcomes, particularly for studies with an older adult or general adult population. In each of these studies, the direction of the outcome was influenced by demographic variables (such as gender) or the type of pet (cat or dog). For example, one general population study determined that women with pets had lower levels of depression whereas men with pets had higher levels of depression [19]. Another example is that pet-owning individuals with severe mental illness had less psychiatric hospitalizations than non-pet owning peers, however, they also had higher levels of substance use [38]. Another reason why a study would be categorized as mixed impact is if mental health was assessed using multiple instruments and yielded conflicting results. For instance, one study indicated that when compared to people without pets, those with pets had no difference in anxiety or stress scores yet had higher depression scores [22].

For the 13 studies that had no impact, most were with the older adult (*n* = 6) and general adult (*n* = 4) population. These studies concluded that when comparing pet ownership to non-pet ownership or when comparing attachment levels, the pet had no correlation with positive or negative mental health. Many of these studies controlled for demographic variables such as age, gender, and socioeconomic status in their statistical models. One challenge to categorizing the studies was that study participants subjectively believed their pets were helpful to their mental health despite what validated measures showed. The inclusion of these biased observations in an attempt to still put a positive spin on the study may reflect the conflict a researcher has in publishing negative results. An additional challenge is that studies that included non-mental health measures (such as physical health) showed that those with pets did better than those without. Expert reviews of pet ownership on cardiovascular health have demonstrated a significant challenge to reach a definitive conclusion of the impact of pet ownership on health based on the current evidence [39].

Five studies demonstrated a clear negative impact between pet ownership and mental health. The sample populations were general (*n* = 2), older adults (*n* = 2), and single adults living alone (*n* = 1). In these studies, pet ownership was associated with higher levels of depression, loneliness, and other psychological symptoms across all demographic variables and type of pet (dog or cat). Again, the challenge to classifying these studies as negative impact suggests that pet ownership causes increased levels of mental health illnesses, when in reality, the studies are about correlation, not causation. There may be other factors that cause the samples in these studies to have worse mental health. As indicated by Mullersdorf et al. [40], the presence of a psychological condition could predispose individuals to become pet owners, making it difficult to truly know if pet ownership causes a negative impact on mental health. These studies, regardless of type of outcome, only indicate association of pet ownership and mental health.

Another challenge in comparing the 54 studies was the difference in methodology and quality of each study. Due to this, our methods did not evaluate the individual and overall power and effect sizes of study results. Quantitative methodologies are warranted in this field, particularly prospective, randomized, double-blind, placebo-controlled intervention trials that are longitudinal in design to provide evidence of the impact of animal ownership over time while eliminating as many extraneous and confounding variables as possible [41]. Ideally, this truly experimental model of pet ownership would include random assignment of companion animals in a closed system to eliminate as many sources of error variance as possible [42]. However, due to the nature of pet ownership being integrated as a part of daily life on a voluntary basis, this experimental model would be difficult to achieve. Perhaps the most compelling of all studies that comes closest to this design was a prospective interventional study in which 71 previous non-pet owners were given a cat or dog; results demonstrated mild benefits in mental health and behavior after 10 months of pet ownership compared to the 26 non-pet owners [43]. While noteworthy, there was lack of randomization, so the pet ownership group consisted of a relatively small number of subjects who were searching for a pet to adopt rather than receiving it on random chance. Regardless, this study still reports an improvement in mental health in this specific population. Future studies should strive to achieve this prospective, controlled, experimental methodology to more compellingly connect pet ownership with mental health.

A quality index attempted to rate the rigor of each study, but the index was subjective and based on questions that could be asked without statistical analysis (e.g., does this study include a comparison population?). The higher the score on the quality index, the more likely the study was scientifically rigorous. The lower the score, the more likely the study was to demonstrate a positive or mixed impact on the pet owner’s mental health. While both previous literature reviews critiqued the rigor of the studies reviewed and remarked upon the consistent methodological flaws, Islam and Towel did not assign objective scores to the 11 studies reviewed. Brooks et al. [17] did assign quality scores to each of the 17 studies reviewed but did not evaluate the impact of the quality of the study on its results. The quality scores in the current review varied across all four outcome categories and did not give any indication of quality impacting the overall outcome. Still, it is important that researchers strive for higher quality research that carries more weight in the question of whether pet ownership truly impacts mental health. Additionally, we recommend that studies be replicated in an attempt to corroborate previous findings, which contribute to the overall understanding of the phenomenon.

Lastly, this study also examined how mental health was evaluated across the studies. For the 54 studies included in this review, 75 different scales (Table 5) were used with many research studies implementing more than one scale (Appendix B). While most of the scales used have been previously validated, the inconsistent use of scales makes comparison of results across studies challenging. While it is common to utilize an instrument that is a validated self-report of depression, it is likely that researchers often utilize other scales because they are investigating other aspects of mental health such as loneliness, stress, and anxiety. Many scales also rely on self-reporting of mental health indicators, which can be affected by inherent bias, especially when completing a survey regarding mental health and pet ownership. To allow for better comparison of future studies, researchers should attempt to use consistent measures of mental health across studies, such as the CES-D [44], which was the most commonly used scale in 13 of the 54 examined studies.

In addition to consistent use of mental health scales across studies, the development of a module for use in wide-scale population surveys with a focus on pet-ownership would benefit future research examining the relationship between pet ownership and health. The Behavioral Risk Factor Surveillance System (BRFSS) [45] is an annual questionnaire administered by the US Centers for Disease Control and Prevention. There are 14 core sections that are administered to all participants and 31 optional modules [45]. None of these modules focuses on pet ownership and the addition of such a module would allow for a more in-depth evaluation of the relationship between pet ownership and health, both mental and physical, across large populations. While pets can play a significant role in the owner’s health, it can be difficult to differentiate the effects of pet ownership from the many other factors that contribute to one’s mental and physical health. The addition of a pet ownership module to the BRFSS would allow researchers to examine the role of pet ownership in tandem with other factors that contribute to health. On a smaller scale (approximately 3000 participants), the General Social Survey (GSS) is a representative survey that monitors trends in opinions, behaviors, and demographics among Americans [46]. Though not a main focus, the GSS does include pet ownership and mental health variables. Including pet ownership allows researchers who study the relationship of ownership with humans to have a large, representative dataset to analyze correlations. For example, a recent study used the GSS 2018 to examine demographics of pet ownership [46]. In their conclusion, the authors of this study indicated that the strengths of using the GSS to study pet ownership characteristics are high quality data, multiple covariates, sound methodology, and easy access [47]. Including pet ownership questions in multi-wave, representative studies would further the work of human animal relationship research.

This systematic review was limited due to only searching two databases and only evaluating research published in English. The majority of studies focused on pet-owners in Western cultures. The human–animal bond may differ across cultures and future studies should include pet-owners in non-Western cultures. However, a large number of articles were identified, and the total number of articles included in final extraction was greater than similar previous systematic reviews. More consistent methods across research that evaluates the relationship between pet ownership and mental health might allow for more extensive comparison of studies.

## 5. Conclusions

Previous research examining the impact of pet ownership on mental health has shown mixed results and the results of this study were the same. While there were more absolute numbers of studies to demonstrate a positive impact (*n* = 17) compared to negative impact (*n* = 5) on mental health, the overall results indicate a much more complicated picture. While 17 of the 54 studies had a clear association of pet ownership and positive mental health, the remaining 37 articles show a mixed association, no association, or a negative association. Comparing these studies is quite challenging due to the number of measures used to assess mental health, the differences in study quality, and the variety of variables that were controlled for. While research studies can be improved by addressing limitations as described, a more comprehensive evaluation of behavior and its association with health outcomes is warranted. We also cannot ignore that mental health is multifactorial. Pet ownership and the resulting human–animal interaction is a single factor; other factors that also contribute to mental health should be examined in large populations of pet-owners and non-pet-owners. The addition of a pet-ownership specific module to the BRFSS, as previously described, would allow for prospective research that can be replicated, and eventually retrospective research, that will also allow for inclusion of other factors that contribute to health.

## Figures and Tables

**Table 1:** Inclusion and exclusion criteria used for evaluation of research articles that examined the relationship between pet ownership and mental health.

Inclusion Criteria	Exclusion Criteria
Original research	Review article/not original research
Pet ownership (dog/cat)	Animal assisted intervention or therapy
Assessment of pet ownership on some classification of mental health	Working/service animal
Accessible through library system	Pet ownership other than dog or cat
Quantitative data reported	Outcome only in animal
Written in English	Not accessible through library system
	Only qualitative data reported
	Not written in English

**Table 2:** At the extraction stage, the following information was used for evaluation of research articles that examined the relationship between pet ownership and mental health.

Information Extracted from Articles
Study purpose
Type of research/Study design
Description of methods
Sample size
Demographics of sample
Type of pet (dog, cat, both)
How mental health diagnosis was obtained (self-report, scale, etc.)
Outcome variables
Mediating and moderating variables
Data analysis type
Main study findings
Type of impact on mental health (positive, mixed, none, negative)

**Table 3:** Outcomes of 41 studies that examined mental health outcomes in pet owners compared to non-pet owners.

Population Studied	Negative Impact	Mixed Impact	Positive Impact	No Impact	Total
Older adult	2	7		5	14 (34%)
Severely mentally ill		1	2		3 (7%)
Children and adolescents		1	4	1	6 (15%)
General	1	4	3	3	11 (27%)
Illness (cancer, back pain, etc.)		1	2	2	5 (12%)
Caregivers		1			1 (2%)
Veterans			1		1 (2%)
Totals	3 (7%)	15 (37%)	12 (29%)	11 (27%)	41

**Table 4:** Outcomes of nine studies that examined mental health outcomes in relationship to the pet owner’s attachment bond with their pet.

Population Studied	Negative Impact	Mixed Impact	Positive Impact	No Impact	Total
Older adult				1	1 (8%)
Children and adolescents			2		2 (15%)
General	1	3	3	1	8 (61%)
Illness (cancer, back pain, etc.)		1			1 (8%)
Adults living alone	1				1 (8%)
Totals	2 (15%)	4 (31%)	5 (38%)	2 (15%)	13

**Table 5:** The scales used across studies to measure mental health.

Category of Mental Health	Measure Used
General mental health	General Mental Health Questionnaire (GHQ) (Versions 12; 30), Brief Symptom Inventory (BSI), Global Assessment of Functioning (GAF), Balanced Measure of Psychological Needs (BMPN), Patient Reported Outcomes (PROMIS), Mental Health Inventory (MHI), Colorado Symptom Inventory (CSI)
Well-being	Dimensions of Well-being (SPF-IL), Psychological Scale of Well-being (PWB), Psychological General Well-being Index (PGWB), Wisconsin Quality of Life Survey (W-QLI), Life Satisfaction Index Psychological Well-being for older adult (LSIA), Life Satisfaction Scale (SWLS), World Health Organization Five Well-being Index (WHO-5), Functional Assessment of Cancer Therapy (FACT-G)
Loneliness	Lubben Social Isolation Scale for Older Adults (LNS-6), De Jong Gierveld Loneliness Scale, UCLA Loneliness Scale (ULS), UCLA Loneliness Revised (ULS-R)
Depression and anxiety	Patient Health Questionnaire (PHQ), Center for Epidemiological Studies Depression Scale (CES-D), Strait-Trait Anxiety Inventory (STAI-T), Screen for Child Anxiety Related Disorders (SCARED-5), Depression Anxiety Distress Scale (DASS), Kessler Psychological Distress (K-10), Beck Depression Inventory (BDI), Hospital Anxiety and Depression (HADS), Brief Symptom Inventory (BSI), Spence Children’s Anxiety Scale (SCAS), Geriatric Depression Scale Short Form (GDS-SF), Goldberg Anxiety and Depression Scale (GADS), Health Anxiety Inventory (HAI), Quick Inventory of Depressive Symptomatology (QIDS), PROMIS Depression, PROMIS Anxiety
Quality of life	Manchester Short Assessment of Quality of Life (MANSA), Health Related Quality of Life (HRQOL), KIDSCREEN-10, World Health Organization Quality of Life (WHOQOL-BREF), Short Form 36 Health Survey Questionnaire (SF-36)
Social support	Interpersonal support evaluation list (ISEL), Jichi Medical School Social Support Scale (JMS-SSS), Psychological Community Integration Scale (CIS-APP-34), Sarason Social Support Questionnaire (SSQSR), Multidimensional Scale of Perceived Social Support (MSPSS), Brief Family Relationship Scale (BFRS), Barrett Lennard Relationship Inventory (BLRI), Networks for Support Scale (SSNS), PROMIS Companionship, PROMIS Emotional Support, Children’s Exposure to Domestic Violence Scale (CEDV), Social Provisions Scale (SPS), Multi-Dimensional Support Scale (MDSS)
Mood and self-regulation	Positive and Negative Affect Schedule (PANAS-SF), Emotional Regulation Questionnaire (ERQ), Modified Differential Emotions Scale (mDES)
Self-esteem, happiness, and life satisfaction	Subjective Fluctuating Happiness Scale (SFHS), Subjective Happiness Scale (SHS), Rosenberg Self Esteem Scale (RSES), Satisfaction with Life Scale (SWLS), Sense of Life Worth Living (IKIGAI), Happiness Index (HI), Life Satisfaction Index Z (LSI-Z), State Trait Hopelessness Scale (STHS)
Stress	Perceived Stress Scale (PSS), Parenting Stress Index (PSI-SF), Humor Stress Questionnaire (HSQ)
Other	Empathy Quotient Questionnaire (EQ), PTSD Checklist (PCL), Eysenck Personality Questionnaire-Revised (EPQ-R), Resilience Research Center Adult Resilience Measure (RRC-ARM), Child and Youth Resilience Measure (CRYM-28), Big Five Inventory (BFI), Personal Attributes Questionnaire (PAQ), Strengths and Difficulties Questionnaire (SDQ), Child Adolescent Bullying Scale (CABS), Alzheimer’s Caregiver Burden Interview (ZBI), Childhood Trauma Questionnaire (CTQ), Stress Salivary Biomarker
Attachment	Lexington Attachment to Pets Scale (LAPS), Short Attachment to Pets Scale (SAPS), Human Animal Bond (HAB), Owner-Pet Relationship Questionnaire (OPRQ), Pet Attachment Questionnaire (PAQ), Barrett-Lennard Relationship Inventory (BLR), CENSHARE Pet Attachment Survey (PAS)

## Data Availability

Data was not generated in this study.

## Appendix A

Article Quality Index	
1	Was the study purpose reported? 1 = yes, 0 = no
2	Did the study design include a comparison group? 1 = yes, 0 = no
3	Was the recruitment method reported? 1 = yes, 0 = no
4	Was the sample size response rate over 50%? 1 = yes, 0 = no (or unreported)
5	Were sample demographics reported? (3 or more demographic categories reported)1 = yes, 0 = no
6	Was the sample representative (not self-selected)? 1 = yes, 0 = no (or not reported)
7	Did mental health diagnosis occur through standardized scale or mental health professionals? 1 = yes, 0 = no
8	Was the validity and/or reliability of scales reported? 1 = yes, 0 = no
9	Was there a study limitation section? 1 = yes, 0 = no

## Appendix B

Following a literature review and data extraction of research articles that examined the relationship between pet ownership and mental health, the following articles were found to meet inclusion and exclusion criteria as outlined in Table 1.

**Author**	**Title**	**Year**	**Sample Size**	**Methods**	**Sample Population**	**Mental Health** **Measurement(s)**	**Findings**	**Article** **Quality** **Index Score (0–9)**
Research with Positive Associations between Pet Ownership and Mental Health								
Serpell, J. [43]	Beneficial effects of pet ownership on some aspects of human health and behaviour	1991	71	-Prospective -Quantitative	General	-GHQ-30	After acquiring a pet, dog-owners demonstrated significant improvement in their GHQ-30 scores during the first six months after acquiring a pet, and moderate at 10-month follow-up. Cat owners demonstrated small and non-statistically significant improvement at six months.	3
Budge, R.C. et al. [48]	Health correlates of compatibility and attachment in human-companion animal relationships	1998	176	-Cross-sectional -Quantitative	General	-AHCS -Pet Attachment Survey -ISEL -MHI	As compatibility in the human–pet relationship increased, so did the physical and mental health and wellbeing for the human. Human–pet compatibility was not associated with levels of social support.	5
Zimolag, U.; Krupa, T. [49]	Pet Ownership as a Meaningful Community Occupation for People With Serious Mental Illness	2009	59	-Cross-sectional -Quantitative	People receiving mental illness treatment	-GAF -EMAS -CIS-APP	Pet owners demonstrated better social community integration than non-pet owners. Pet owners may also engage in more meaningful activity and have higher psychological community integration than non-pet owners.	6
McConnell, A.R. et al. [50]	Friends with benefits: on the positive consequences of pet ownership	2011	217	Prospective, cross section -Quantitative	General	-CES-D -UCLA -RSES -SHS	Pets can serve as effective social resources for their owners and positive connections with pets are correlated with positive attachment styles, personality traits, and self-esteem generally and when facing social rejection.	4
Black, K. [51]	The Relationship Between Companion Animals and Loneliness Among Rural Adolescents	2012	293	-Cross-sectional -Quantitative	Adolescents	-ULS -CABS -SSQSR	Pet owning adolescents had significantly lower loneliness scores and there was an inverse relationship between level of bond with pet and levels of loneliness.	8
Stern, S.L. et al. [52]	Potential Benefits of Canine Companionship for Military Veterans with Posttraumatic Stress Disorder (PTSD)	2013	30	-Cross-sectional -Quantitative	Military Veterans	-BDI -LAPS -PCL	Since adopting their dog, veterans self-reported feeling calmer, less lonely, less depressed, and less worried about their and their family’s safety. Veterans did not report less PTSD symptomatology since adopting their dog.	6
Wright, H. et al. [53]	Pet Dogs Improve Family Functioning and Reduce Anxiety in Children with Autism Spectrum Disorder	2015	70	-Cross-sectional -Quantitative	Children with ASD and their families	-BFRS -SCAS	Family functioning improved and child anxiety decreased in the dog-owning group compared to the non-dog owning group.	6
Gadomski, A.M. et al. [54]	Pet Dogs and Children’s Health: Opportunities for Chronic Disease Prevention?	2015	643	-Cross-sectional -Quantitative	Children	-SCARED-5 -SDQ -PHQ-2	Having a pet dog in the home was associated with a decreased probability of childhood anxiety.	5
Lem, M.; Coe et al. [55]	The Protective Association between Pet Ownership and Depression among Street-involved Youth: A Cross-sectional Study	2016	190	-Cross-sectional -Quantitative	Children and adolescents	-CES-D	Pet ownership among street youth was associated with lower levels of depression.	7
Bos, E.H. et al. [29]	Preserving Subjective Wellbeing in the Face of Psychopathology: Buffering Effects of Personal Strengths and Resources	2016	2411	-Cross-sectional -Quantitative	General	-MANSA -HI -PWB -SPF-IL -DASS -QIDS -PANAS -HSQ -EQ	Owning a pet and/or having a partner protected study participants’ wellbeing even when psychological distress symptoms were present.	6
Hall, S.S. et al. [56]	The long-term benefits of dog ownership in families with children with autism	2016	37	-Longitudinal -Mixed	Children with ASD and their families	-PSI-SF -LAPS	Families of autistic children who had acquired a pet dog demonstrated improved family functioning and reduced parental stress in comparison to control group families who did not acquire a pet dog.	8
Marsa-Sambola, F. et al. [57]	Quality of life and adolescents’ communication with their significant others (mother, father, and best friend): the mediating effect of attachment to pets	2017	2262	-Cross-sectional -Quantitative	Adolescents	-KIDSCREEN-10 Index -SAPS	Higher attachment to pet dog/cat was associated with better quality of life. Attachment to pets may also enhance communication with parents and best friends.	6
Muldoon, A.L. et al. [58]	A Web-Based Study of Dog Ownership and Depression Among People Living With HIV	2017	199	-Cross-sectional -Quantitative	People with a physical illness	-CES-D10 -RRC-ARM -CYRM-28	Non-current dog ownership among research participants was significantly and positively associated with depression with non-current dog owners being three times more likely to report symptoms of depression compared with current dog owners.	8
Wu, C.S.T. et al. [59]	The Association of Pet Ownership and Attachment with Perceived Stress among Chinese Adults	2018	288	-Cross-sectional -Quantitative	General	-PSS -CABS	Higher levels of pet attachment are associated with lower levels of perceived stress among pet owners. Dog owners report being more attached to their pet than other types of pet owners.	7
Powell, L. et al. [60]	Companion dog acquisition and mental well-being: a community-based three-arm controlled study	2019	71	-Prospective -Quantitative	General	-ULS -PANAS -K-10	Acquiring a dog was associated with lower levels of loneliness at three-month and eight-month follow up.	6
Carr, E.C.J. et al. [61]	Evaluating the Relationship between Well-Being and Living with a Dog for People with Chronic Low Back Pain: A Feasibility Study	2019	56	-Cross-sectional -Mixed methods	People with a physical illness	-HRQOL -WHO-5 -PROMIS anxiety SF4 -PROMIS depression SF4 -ULS -SSNS -PROMIS Companionship scale -PROMIS Emotional support scale -LAPS -HAB	Dog owners reported fewer depression and anxiety symptoms over the last week before the survey than the non-dog owners.	5
Yolken, R. et al. [28]	Exposure to household pet cats and dogs in childhood and risk of subsequent diagnosis of schizophrenia or bipolar disorder	2019	1371	-Cross-sectional -Qualitative	People receiving mental illness treatment	N/A	Exposure to a pet dog during the first 12 years of life was associated with a decreased hazard of having a subsequent diagnosis of schizophrenia.	5
Research with Mixed Associations between Pet Ownership and Mental Health								
Siegel, J.M. [62]	Stressful life events and use of physician services among the elderly: the moderating role of pet ownership	1990	938	-Prospective -Mixed	Elderly	-LNS -CES-D	Elderly respondents with stressful life events made fewer visits to the physician if they had a pet dog. The presence of a dog was not associated with lower levels of depression.	6
Gulick, E.E.; Krause-Parello, C. A. [63]	Factors related to type of companion pet owned by older women	2012	159	-Cross-sectional -Quantitative	Elderly (females)	-PGWB -ULS	Women with dogs reported higher general health, vitality, and total well-being but worse levels of depression than women with cats.	6
Fritz, C.L. et al. [64]	Companion animals and the psychological health of Alzheimer patients’ caregivers	1996	244	-Cross-sectional -Quantitative	Caregivers	-ZBI -LSI-Z -GDS -LAPS	Stress was less for pet owning younger male and female caregivers of cognitively impaired adults but not for older pet owning female caregivers.	5
Tower, R.B.; Nokota, M. [19]	Pet companionship and depression: results from a United States Internet sample	2006	2291	-Cross-sectional -Quantitative	General	-CES-D	Unmarried women who live with a pet had the fewest depressive symptoms and unmarried men who live with a pet had the most.	6
Wisdom, J.P.; Saedi, G.A.; Green, C.A. [38]	Another Breed of “Service” Animals: STARS Study Findings About Pet Ownership and Recovery From Serious Mental Illness	2009	177	Prospective -Longitudinal -Mixed Methods	People with serious mental illness	-CSI -W-QLI	Pet owners were more likely to have affective versus psychotic diagnosis, were more likely to have a comorbid substance abuse disorder and were more likely to live with someone. They also had fewer hospitalizations.	4
Cline, K.M.C. [65]	Psychological Effects of Dog Ownership: Role Strain, Role Enhancement, and Depression	2010	201	-Cross-sectional -Quantitative	General	-CES-D	Dog ownership had no direct impact on depression. Dog ownership was associated with greater wellbeing for women and those who are unmarried.	7
Ramirez, M.T.G.; Hernandez, R.L. [66]	Benefits of dog ownership: Comparative study of equivalent samples	2014	602	Prospective- Cross-Sectional -Quantitative (Snowball sampling)	General	-SWLS -SHS -PHQ -PSS -SFHS	Dog owners’ scores were significantly lower for psychosomatic symptoms and stress and were higher for better mental health, however, there were no differences between groups for happiness and life satisfaction.	5
Bradley, L.; Bennett, P.C. [22]	Companion-Animals’ Effectiveness in Managing Chronic Pain in Adult Community Members	2015	173	-Cross-sectional -Mixed methods	People with physical illness	-DASS-21	There was no relationship between companion animal ownership and stress or anxiety, however, owners had higher levels of depression than non-owners. Depression among those who perceived their animal as more friendly was lower and for those who perceived their animal as more disobedient stress was higher.	5
Girardi, A.; Pozzulo, J.D. [24]	Childhood Experiences with Family Pets and Internalizing Symptoms in Early Adulthood	2015	318	-Cross-sectional -Quantitative	General	-LAPS -CTQ -CEDV -STAI-T -BDI	Participants who were exposed to pet aggression in childhood and reported medium level bonds with animals also reported more depression and anxiety symptoms in early adulthood. Those who were not exposed to pet aggression reported fewer internalizing symptoms.	7
Bennett, P.C. et al. [67]	An Experience Sampling Approach to Investigating Associations between Pet Presence and Indicators of Psychological Wellbeing and Mood in Older Australians	2015	68	-Prospective Experience sampling over 7 days -Quantitative	Elderly	-DASS -SPS -ULS-R	There was not a difference between pet-owners and non-pet-owners in mental health outcomes, however, for pet owners, level of pet presence in daily activities was associated with better mental health outcomes.	6
Branson, S.M. et al [68].	Depression, loneliness, and pet attachment in homebound older adult cat and dog owners	2017	39	-Cross-sectional -Quantitative	Elderly	-GDS-SF -ULS-R	Cat owners reported fewer depressive symptoms than dog owners, especially for men, but the differences in levels of depressive symptoms between dog and cat owners was small.	6
Mueller, M.K. et al. [69]	Human-animal interaction as a social determinant of health: descriptive findings from the health and retirement study	2018	1657	-Retrospective -Cross-sectional -Quantitative	Elderly	- Created measures	Pet ownership was positively correlated with reporting depression in lifetime, however, there was no difference in self-reported depression in the last week between pet owners and non-owners.	6
Carr, D.C. et al. [70]	Typologies of older adult companion animal owners and non-owners: moving beyond the dichotomy	2019	1179	-Cross-sectional -Quantitative Data was collected from the Health a Retirement Study	Elderly	-CES-D -BFI	Five clusters of owners and four clusters of non-owners were identified with varying mental health outcomes. Pet owners were higher in neuroticism and lower in extraversion.	5
Liu, S.X. et al. [30]	Is Dog Ownership Associated with Mental Health? A Population Study of 68,362 Adults Living in England	2019	68,362	-Repeated cross-sectional survey running in annual thematic cycles -Quantitative	General	-GHQ-12	Single dog owners were more likely to demonstrate higher levels of short-term psychological distress. Dog owners with partners had lower levels of self-reported mental illness.	7
Ingram, K.M.; Cohen-Filipic, J. [20]	Benefits, challenges, and needs of people living with cancer and their companion dogs: An exploratory study	2019	122	-Cross-sectional -Mixed methods	People with physical illness	-CES-D -FACT-G -LAPS	The human–pet bond was not directly linked with well-being. Depressive symptoms depended on cancer treatment status and level of bond with those having completed treatment and had a stronger bond reported fewer depressive symptoms. For continuing treatment stronger bonds was positively correlated with depression.	5
Min, K.D. et al. [71]	Owners’ Attitudes toward Their Companion Dogs Are Associated with the Owners’ Depression Symptoms-An Exploratory Study in South Korea	2019	654	-Cross-sectional -Quantitative	General	-CES-D	Those respondents who had a negative view of their pets also were more likely to report the presence of depression. Those who had a more positive view of their pet were less likely to report depression.	5
Hajek, A.; Konig, H.H. [23]	How do cat owners, dog owners and individuals without pets differ in terms of psychosocial outcomes among individuals in old age without a partner?	2020	1160	-Longitudinal -Cross-sectional -Quantitative	Elderly	-CES-D -De Jong Gierveld Loneliness Scale	Dog owners were less socially isolated than non-pet owners, however this was not true for cat owners. Pet-owning women also reported less loneliness, whereas loneliness did not differ between pet-owning and non-pet-owning men.	8
Teo, J.T.; Thomas, S. J. [72]	Psychological Mechanisms Predicting Wellbeing in Pet Owners: Rogers’ Core Conditions versus Bowlby’s Attachment	2019	298	-Cross-sectional -Quantitative	General	-DASS-21 -BSI -WHO QOLBREF -OPRQ -PAQ -BLRI	Pet owners and non-pet owners did not significantly differ in terms of QOL or psychopathology. However, in pet owners, secure pet attachments were associated with lower psychological distress and psychopathology. Differences in wellbeing is related to qualities of individual human–pet relationships.	7
Endo, K. et al. [73]	Dog and Cat Ownership Predicts Adolescents’ Mental Well-Being: A Population-Based Longitudinal Study	2020	2584	-Prospective cohort study -Quantitative and qualitative	Adolescents	-WHO-5	Dog ownership at age 10 predicted better well-being at age 12 compared to no dog ownership. Cat ownership at age 10 predicted worse well-being at age 12 compared to no cat ownership.	4
Research with Negative Associations between Pet Ownership and Mental Health								
Parslow, R.A. et al. [74]	Pet ownership and health in older adults: Findings from a survey of 2551 community-based Australians aged 60–64	2005	2551	-Cross-sectional -Quantitative	Elderly	-SF-12 -GADS -PANAS -EPQ-R	Those with pets have poorer mental and physical health and use more pain relief medication than those without pets. Further, our study suggests that those with pets are less conforming to social norms as indicated by their higher levels of psychoticism.	6
Mullersdorf, M. et al. [40]	Aspects of health, physical/leisure activities, work and socio-demographics associated with pet ownership in Sweden	2010	39,995	Retrospective- Cross-sectional -Quantitative	General	-Aspects of health and mental health on a five-point Likert scale	“Pet owners in this study reported poorer mental health than non-pet owners. However, the authors suggest that the increase in depression or feelings of loneliness might predispose people to buying a pet.”	7
Antonacopoulos, N.M.D.; Pychyl, T.A. [13]	An Examination of the Potential Role of Pet Ownership, Human Social Support and Pet Attachment in the Psychological Health of Individuals Living Alone	2010	132	-Cross-sectional -Mixed Methods	General (adults living alone)	-MSPSS -LAPS -CES-D -ULS	“High attachment to pets predicted significantly higher scores on loneliness and depression. Our findings emphasize the complexity of the relationship between pet ownership and psychological health and suggest that pet ownership may not be beneficial for the psychological health of all individuals living alone.”	7
Peacock, J. et al. [12]	Mental health implications of human attachment to companion animals	2012	150	-Cross-sectional -Quantitative	General	-BSI-18 -MDSS -CEN SHARE PAS -OPRQ	“Human–animal relationships are associated with increased reports of psychological symptomatology. This study adds to the body of evidence that suggests that to understand human-animal relationships and their impact on well-being, it is pivotal to assess what the relationship symbolizes for an individual.”	6
Sharpley, C.et al. [75]	Pet ownership and symptoms of depression: A prospective study of older adults	2019	5334	-Longitudinal -Cross-sectional -Quantitative	Elderly	-CES-D	“In conclusion, the present results indicate that an increase in depressive symptoms is associated with higher odds of dog ownership in community-dwelling older people, but provide no evidence of a protective effect of pet ownership on changes in depressive symptoms over time.”	6
Research with No Associations between Pet Ownership and Mental Health								
Raina, P. et al. [76]	Influence of companion animals on the physical and psychological health of older people: an analysis of a one-year longitudinal study	1999	995	-Longitudinal -Cross-Sectional	Elderly	-LAPS -Reported levels of satisfaction regarding mental health, happiness, and relationships	“No statistically significant direct association was observed between pet ownership and change in psychological wellbeing However, pet ownership significantly modified the relationship between social support and the change in psychological well-being over a 1-year period.”	4
El-Alayli, A. et al. [77]	Reigning cats and dogs: A pet-enhancement bias and its link to pet attachment, pet-self similarity, self-enhancement, and well-being	2006	70	Prospective -Quantitative	General	-PAS -CABS -SWLS -PANAS -SHS	“A secondary objective of this research was to examine whether psychological well-being was related to pet enhancement, pet attachment, and pet–self similarity. We found no evidence suggesting a linear relationship between pet attachment and psychological well-being.”	6
Wells, D.L. [78]	Associations between pet ownership and self-reported health status in people suffering from chronic fatigue syndrome	2009	193	Cross-sectional	People with physical illness	-GHQ-12 -SF36	Overall, findings suggest no statistically significant association between pet ownership and self-reported health in people with CFS. Nonetheless, people suffering from this condition believe that their pets have the potential to enhance quality of life.	6
Nagasawa, M.; Ohta, M [79].	The influence of dog ownership in childhood on the sociality of elderly Japanese men	2010	220	-Cross-sectional -Quantitative	Elderly	-IKIGAI -ULS-R -JMS-SSS	The effect of dog ownership on the mental condition of an elderly Japanese male may or may not be related to the early childhood dog ownership.	6
Rijken, M.; Van Beek, S. [80]	About Cats and Dogs Reconsidering the Relationship Between Pet Ownership and Health Related Outcomes in Community-Dwelling Elderly	2011	1410	-Prospective -Cross-sectional -Quantitative	Elderly	-GHQ-12 -ULS	Associations between pet ownership and the frequency of social contacts or feelings of loneliness were not found. Having a dog increased the likelihood of being healthy/active, whereas having a cat showed the opposite.	8
Ramirez, M.T.G., et al. [66]	Benefits of dog ownership: Comparative study of equivalent samples	2014	602	-Prospective survey -Quantitative	general	-SWLS -SHS -PHQ -PSS -SF-36	Dog owners had lower stress than non-dog owners, but there was no difference in overall mental health or happiness.	5
Enmarker, I. et al. [81]	Depression in older cat and dog owners: the Nord-Trondelag Health Study (HUNT)-3	2015	12,093	-Cross-sectional -Mixed methods	Elderly	-HADS-d	When comparing pet owners and non-pet owners, self-reported symptoms of depression in older women do not change based on ownership.	7
Bao, K.J.; Schreer, G. [82]	Pets and Happiness: Examining the Association between Pet Ownership and Wellbeing	2016	262	-Cross-sectional -Quantitative	general	-SHS -SWLS -mDES -ERQ -BMPN -BFI	Participants who owned pets and those who did not own pets did not appear to be very different in terms of wellbeing, personality, happiness, positive emotions, or negative emotions. Dog owners were happier than cat owners.	7
Miles, J.N.V. et al. [83]	A Propensity-Score-Weighted Population-Based Study of the Health Benefits of Dogs and Cats for Children	2017	5191	-Retrospective -Cross-sectional	Children	-GHQ-12 -SF-36	When variables related to child development were controlled for, there was no evidence of a positive impact of pet ownership on child mental health.	4
Batty, G.D. et al. [84]	Associations of pet ownership with biomarkers of ageing: population based cohort study	2017	8785	-Prospective -Quantitative	Elderly	-CES-D	There was no evidence of a clear association of any type of pet ownership with depressive symptoms	6
Dunn, S.L. et al. [85]	Dog Ownership and Dog Walking The Relationship With Exercise, Depression, and Hopelessness in Patients With lschemic Heart Disease	2018	122	-Prospective -Quantitative	Physical illness	-PHQ-9 -STHS	No differences in levels of hopelessness between the groups. Dog owners were more depressed until adjusting for age and sex, then no significant differences between dog owners and non-dog owners.	8
Zijlema, W.L. et al. [86]	Dog ownership, the natural outdoor environment and health: a cross-sectional study	2019	3586	-Cross-sectional -Quantitative	General	-SF-36	There was no indication for an association between dog ownership and mental health in groups with high or low access to natural outdoor environment (NOE) and with high or low residential surrounding greenness on the whole.	6
Branson, S.M. et al. [87]	Biopsychosocial Factors and Cognitive Function in Cat Ownership and Attachment in Community-dwelling Older Adults	2019	96	-Cross-sectional -Quantitative	Elderly	-LAPS -PSS -ULS -GDS-SF -Stress Salivary Biomarker	No associations with the biopsychosocial and cognitive measures. No link between the level of pet attachment and loneliness and depression.	7
